# Differences in Lower Extremity and Trunk Kinematics between Single Leg Squat and Step Down Tasks

**DOI:** 10.1371/journal.pone.0126258

**Published:** 2015-05-08

**Authors:** Cara L. Lewis, Eric Foch, Marc M. Luko, Kari L. Loverro, Anne Khuu

**Affiliations:** 1 Department of Physical Therapy & Athletic Training, Boston University, Boston, MA, United States of America; 2 Nutrition, Exercise, and Health Sciences, Central Washington University, Ellensburg, WA, United States of America; Emory University School Of Medicine, UNITED STATES

## Abstract

The single leg squat and single leg step down are two commonly used functional tasks to assess movement patterns. It is unknown how kinematics compare between these tasks. The purpose of this study was to identify kinematic differences in the lower extremity, pelvis and trunk between the single leg squat and the step down. Fourteen healthy individuals participated in this research and performed the functional tasks while kinematic data were collected for the trunk, pelvis, and lower extremities using a motion capture system. For the single leg squat task, the participant was instructed to squat as low as possible. For the step down task, the participant was instructed to stand on top of a box, slowly lower him/herself until the non-stance heel touched the ground, and return to standing. This was done from two different heights (16cm and 24cm). The kinematics were evaluated at peak knee flexion as well as at 60° of knee flexion. Pearson correlation coefficients (r) between the angles at those two time points were also calculated to better understand the relationship between each task. The tasks resulted in kinematics differences at the knee, hip, pelvis, and trunk at both time points. The single leg squat was performed with less hip adduction (p ≤ 0.003), but more hip external rotation and knee abduction (p ≤ 0.030), than the step down tasks at 60° of knee flexion. These differences were maintained at peak knee flexion except hip external rotation was only significant in the 24cm step down task (p ≤ 0.029). While there were multiple differences between the two step heights at peak knee flexion, the only difference at 60° of knee flexion was in trunk flexion (p < 0.001). Angles at the knee and hip had a moderate to excellent correlation (r = 0.51–0.98), but less consistently so at the pelvis and trunk (r = 0.21–0.96). The differences in movement patterns between the single leg squat and the step down should be considered when selecting a single leg task for evaluation or treatment. The high correlation of knee and hip angles between the three tasks indicates that similar information about knee and hip kinematics was gained from each of these tasks, while pelvis and trunk angles were less well predicted.

## Introduction

With increasing interest in injury prevention, functional “screening” tests have become more commonly used to evaluate the movement system [1]. For these tests, a clinician observes as the participant performs a task and notes any abnormal movement patterns. Two frequently used functional tasks for the lower extremity are the single leg squat [2–5] and the step down [6–9]. In the single leg squat task, the participant stands on one leg and squats either to a predetermined knee angle [10–17] or as far as possible [4,18–21]. In the step down task, the participant stands on the top of a box or step where the height is either fixed [6–8,22,23], or adjusted based on participant height [24–26] or tibial length [27]. In a controlled manner, the participant lowers him/herself until the non-stance heel touches the ground and then returns to upright standing on top of the box.

Although the single leg squat is more common, both tasks have been found to be reliable [4,6,23,28], valid [3,29], and useful in identifying abnormal movement patterns in the trunk and lower extremities. For example, increased ipsilateral trunk lean [30], contralateral pelvic drop [30], hip adduction [30,31], and knee abduction [30] have been noted during the single leg squat in patients with patellofemoral pain (PFP) in comparison to controls. During the step down task, participants with PFP have increased ipsilateral trunk lean, contralateral pelvic drop, hip adduction, and knee abduction compared to controls [25]. Females with PFP have greater peak hip internal rotation compared to controls [32]. Furthermore, the increased hip adduction, hip internal rotation, and knee abduction have been associated with higher levels of pain and reduced function in patients with PFP [24]. While the research thus far has primarily focused on PFP [5,6,21,22,24,25,27,32–37] and anterior cruciate ligament (ACL) injury [15,38], the clinical value of these tasks is recognized for patients with hip pain as well [2].

Despite the similarities between tasks, there are differences which may affect the performance of the task. During the single leg squat task, the goal is to keep the non-stance limb off the ground. In contrast, the goal of the step down task is to touch the ground with the heel of the non-stance limb. Given the differences in goals, it is unclear how the normal lower extremity kinematics during these tasks compare between tasks and additionally if there are differences when the step down is performed from different step heights. Identifying potential kinematic differences between the single leg squat and step down tasks, as well as step down tasks of different heights, could provide clinicians with valuable insight that improve the evaluation and treatment process. Therefore, the objective of this study was to identify sagittal, frontal, and transverse plane kinematic differences that may exist at the knee, hip, pelvis, and trunk between the single leg squat and step down tasks in healthy participants. Correlations were also tested to better understand the relationship between each task.

## Materials and Methods

### Participants

An a priori power analysis was conducted to determine the sample size. Based on pilot data on hip adduction angle (a primary variable of interest), an effect size of 1.00 was estimated. Given an alpha of 0.05 and beta of 0.2, a minimum of 10 participants was needed to detect within-subject differences between tasks. A convenience sample of fourteen young healthy adults (females = 10, males = 4; age 23.9 ± 2.0 years; height 1.70 ± 0.12 m; mass 68.7 ± 14.0 kg; UCLA activity score 8.5 ± 1.5) participated in this study.

### Ethics Statement

The Institutional Review Board of Boston University approved this study. All participants provided their written informed consent prior to participation.

### Experimental Protocol

**Fig 1:**
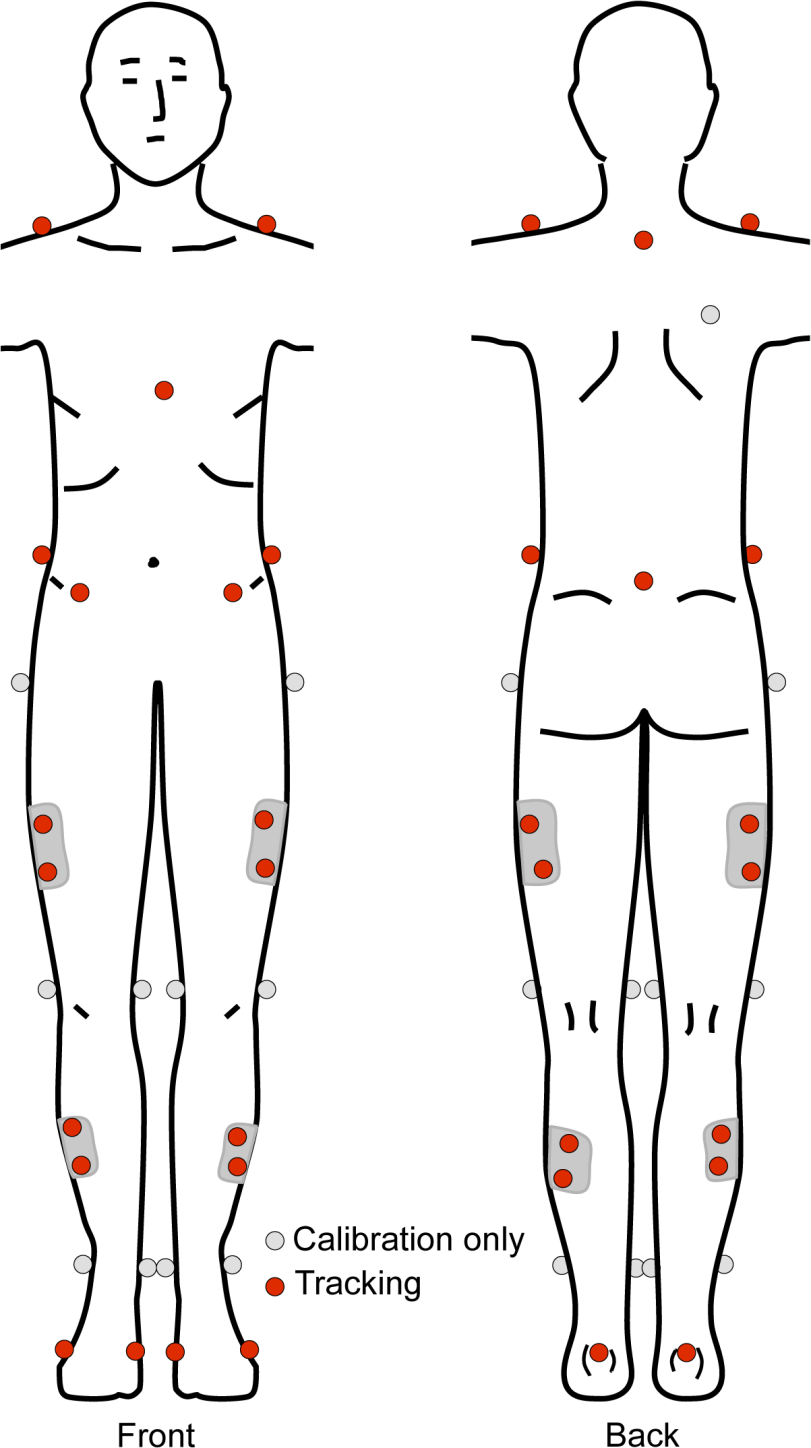
Representation of the 42 spherical, retro-reflective markers placed bilaterally over landmarks on the lower extremity, pelvis and trunk.

Participants wore spandex shorts, a form-fitting shirt, and their own exercise shoes. Forty-two spherical, retro-reflective markers (14mm diameter) were placed, bilaterally, on the lower extremity, pelvis, and trunk (Fig 1). Specifically, markers were placed over the following landmarks: acromion processes, xiphoid process, spinous process of the seventh cervical vertebra (C7), superior aspects of the iliac crests, anterior superior iliac spines, sacrum (midpoint between the posterior superior iliac spines), greater trochanters, lateral and medial femoral epicondyles, lateral and medial malleoli, posterior aspect of the calcanei, and first and fifth metatarsal heads. Plastic shells that contained four, non-collinear markers each were positioned laterally over the thigh and shank [39]. Marker shells were attached to the lower extremity segments via neoprene wraps and hook and loops fasteners. Once the markers were placed over the appropriate anatomical landmark, a static calibration trial was recorded. Following the static trial, the medial knee and ankle markers were removed so they would not encumber participants during the movement trials.

**Fig 2:**
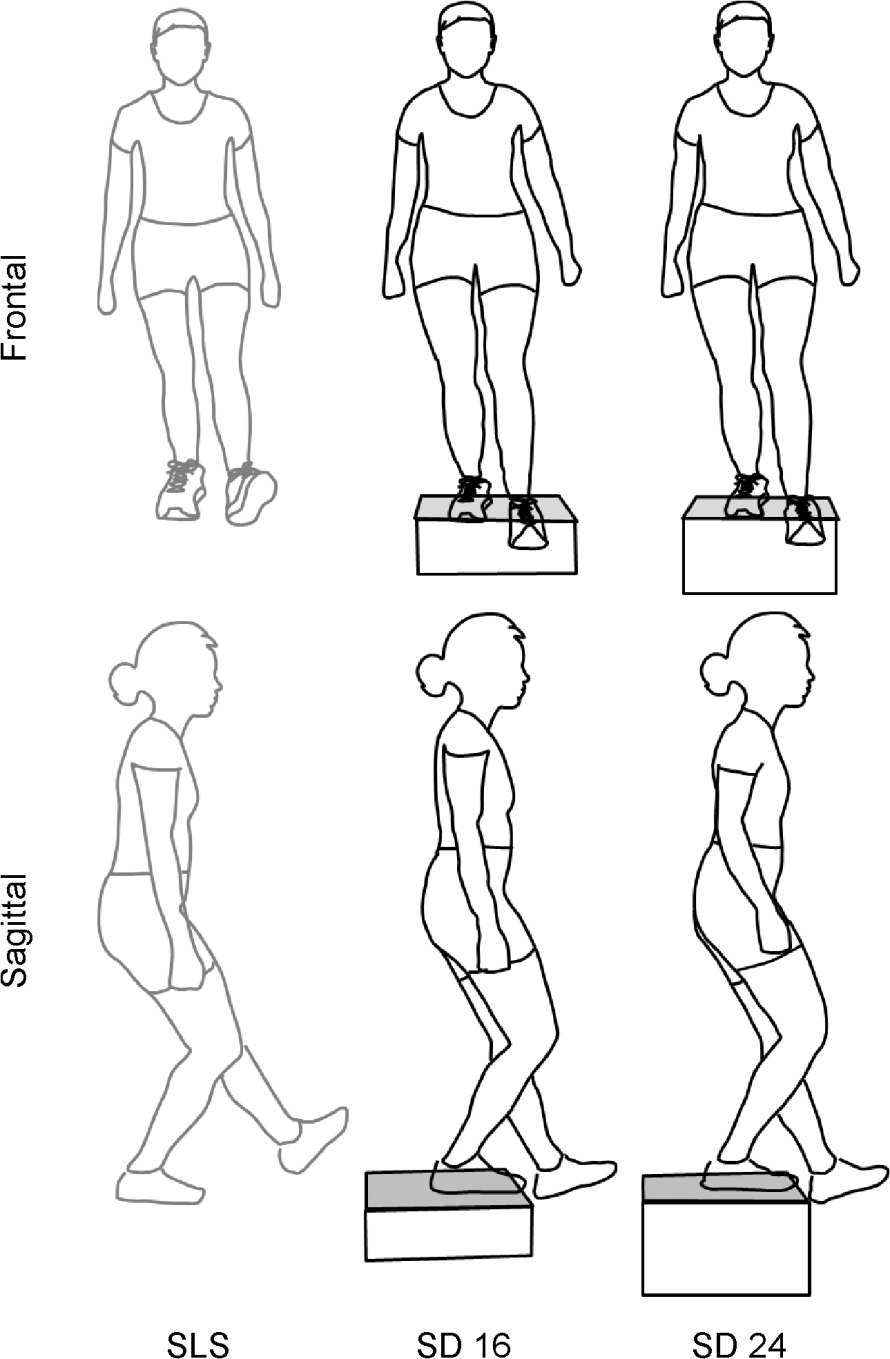
Pictorial representation of a participant performing the single leg squat task (SLS) and step down task from a 16 cm height (SD16) and from a 24 cm height (SD24) at 60° of knee flexion.

Marker motion was recorded using motion capture system (Nexus, Vicon Motion Systems Ltd, Centennial, CO) with ten Vicon MX-T20 cameras sampling at 100 Hz. Each camera was calibrated to have less than 0.15 mm residual error. Participants performed three different single leg weight bearing tasks (Fig 2) in the following order: step down from a 16 cm step (SD16), step down from a 24 cm step (SD24), and single leg squat (SLS). Order was not randomized as this was part of a larger clinical study. For each trial of each task, the starting and ending position was standing on both legs with feet in a self-selected position. For the step down tasks, participants stood with both feet on top of a wooden box. From the starting position, they were instructed to stand on one leg, lower the non-stance limb until the heel lightly touched the floor and then return to standing with both feet on the box. For the single leg squat task, participants were instructed shift their weight onto one leg, squat as low as possible with their non-stance limb extended anteriorly, and return to standing on both legs. The position of the non-stance limb was selected to be similar to the step down task. Participants had an opportunity to practice each task. Participants were given approximately 10 seconds between each single trial of each task, and approximately two to three minutes between tasks. The same leg was always tested first. A metronome set at 60 beats per minute was used to help standardize movement speed. Participants were instructed to try to move “down on a beat and up on a beat.” Participants were given feedback to help maintain a consistent movement speed for each individual trial; however, strict adherence to the metronome was not enforced. Upper extremities had to be maintained either at their sides or out to the side. Trials in which subjects lost their balance or used their upper extremities for support on the surrounding bars were recollected. Five trials were collected on each lower extremity for each task. Only the data during right stance was analyzed for this study.

### Data Processing

Data were processed in Visual3D (C-Motion, Inc., Germantown, MD) as previously described by this laboratory [40,41]. Marker trajectories were low-pass filtered at 6 Hz using a 4th order Butterworth filter [42]. Knee and hip joint angles were defined as the angle between the distal segment and the proximal segment. Joint angles were determined using a Visual3D hybrid model with a Cardan X-Y-Z (mediolateral, anteroposterior, vertical) rotation sequence [43]. The pelvis was defined using the CODA model [44]. Pelvis and trunk segment angles were determined with respect to the global coordinate system. Motion of the pelvis about the mediolateral, anteroposterior, and vertical axes was described as tilt, drop, and rotation, respectively. Joint and segment angles were extracted using custom written code (MATLAB, MathWorks, Natick, MA) at two time points: at peak knee flexion and when the knee of the stance limb first reaches 60° of flexion during the descent phase, and averaged for each task.

### Statistical Analysis

Separate repeated measures analyses of variance (ANOVA) were performed for each dependent variable with task (SD16, SD24 and SLS) as the within-subject factor. The dependent variables were the knee, hip, pelvis, and trunk angles of the right stance limb in the sagittal, frontal, and transverse planes at peak knee flexion and at 60° of knee flexion. The Mauchly’s Test of Sphericity was used to determine if the variances of the differences between all combinations of the three single leg tasks were equal. If the assumption of sphericity was violated, then the Greenhouse-Geisser correction was applied. When a significant main effect was identified for task, post-hoc paired *t*-tests with a Bonferonni correction were used to determine where any significant differences existed. Effect sizes (ES) were computed using Cohen’s d, and can be interpreted as small, medium, and large based on ES values of 0.2, 0.5, and 0.8 respectively [45]. Additionally, Pearson correlation coefficients (r) were calculated to test the linear relationship for each variable between the three tasks. P-values were corrected for the multiple comparisons of each variable. Correlation coefficients can be interpreted as little to no relationship (r = 0.00–0.25), fair relationship (r = 0.25–0.50), moderate to good relationship (r = 0.50–0.75), and good to excellent relationship (r > 0.75) [46]. All analyses were run in IBM SPSS Statistics version 20 (IBM Corporation, Armonk, NY) with an alpha level of 0.05.

## Results

**Fig 3:**
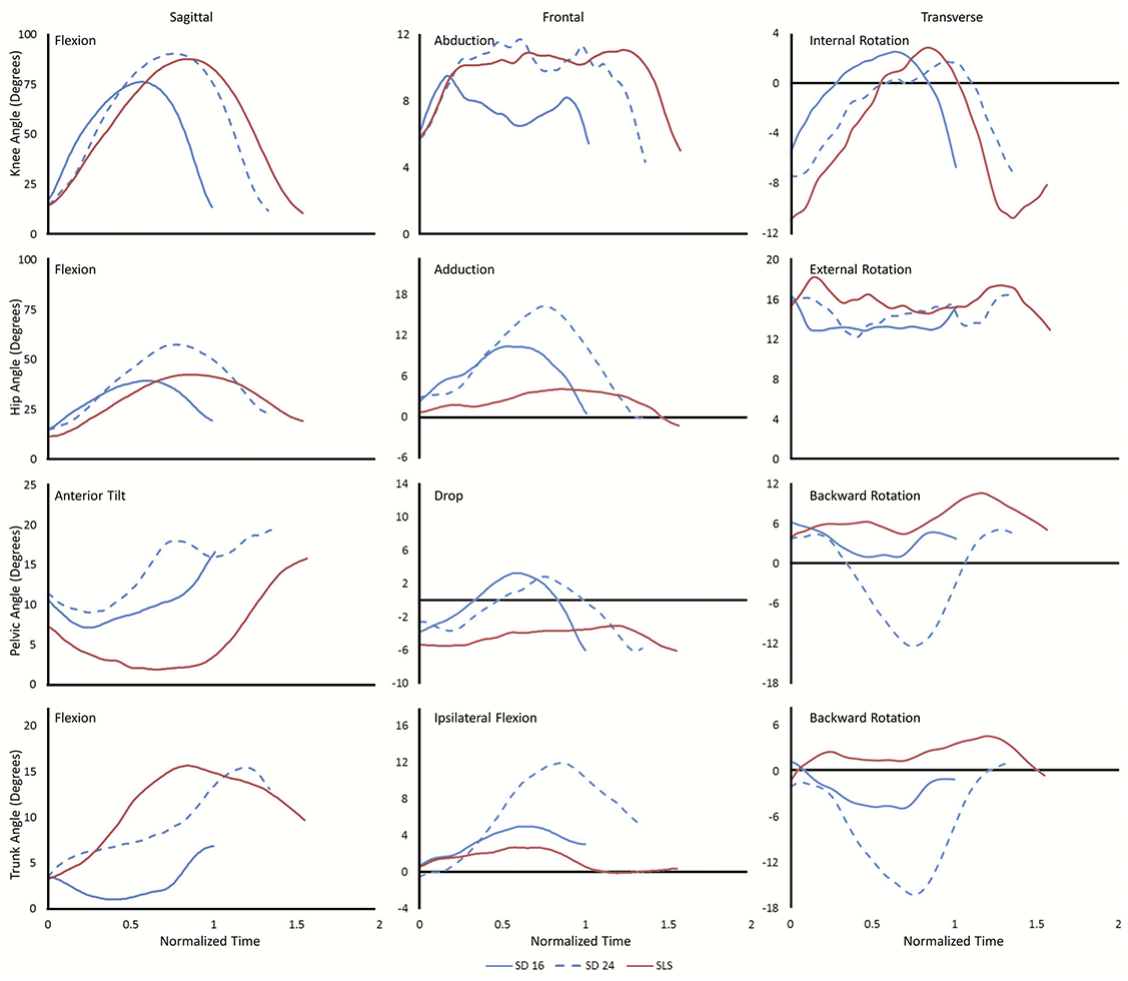
Average knee, hip, pelvis and trunk angles from a representative participant performing each of the three tasks. As the task durations were not equal, data were normalized to maintain the time differences between tasks for presentation purposes only.

The three single leg tasks resulted in kinematic differences at the knee, hip, pelvis and trunk (Fig 3). Repeated measures ANOVAs were significant (p < 0.05) for all dependent variables at both time points except for knee and hip flexion at 60° of knee flexion (Table 1) and thus were followed with post-hoc tests with Bonferroni correction (Table 2). The Greenhouse-Geisser correction was applied to trunk extension at peak knee flexion and to knee abduction, knee rotation, hip flexion, pelvic tilt and drop, and trunk extension at 60˚ of knee flexion. All correlations were positive (Table 3).

**Table 1:** Mean and standard deviation (SD) for each variable (degrees) for each task at the two time points.

	SLS		SD16		SD24		RM ANOVA	
Movement	Mean	(SD)	Mean	(SD)	Mean	(SD)	F	p
**At Peak Knee Flexion**								
Knee Flexion	84.4	(7.3)	72.8	(5.3)	86.9	(8.3)	38.1	**<0.001**
Knee Abduction	4.7	(4.1)	2.0	(4.1)	0.8	(3.4)	14.9	**<0.001**
Knee Internal Rotation	1.0	(7.0)	1.3	(7.7)	2.3	(8.0)	1.9	0.171
Hip Flexion	48.7	(13.7)	42.8	(7.7)	56.9	(9.7)	21.4	**<0.001**
Hip Adduction	8.6	(7.2)	13.7	(7.5)	17.6	(7.4)	40.6	**<0.001**
Hip External Rotation	8.0	(5.3)	4.3	(5.3)	6.4	(5.5)	13.8	**<0.001**
Pelvic Anterior Tilt	3.7	(10.5)	11.3	(6.6)	15.5	(8.1)	45.7	**<0.001**
Pelvic Drop	-1.8	(2.6)	3.1	(2.8)	4.6	(3.5)	56.1	**<0.001**
Pelvic Backward Rotation	-1.2	(7.1)	-4.3	(4.9)	-11.3	(6.7)	21.3	**<0.001**
Trunk Flexion	14.7	(9.5)	4.5	(6.0)	9.6	(5.3)	11.5	**0.003**
Trunk Ipsilateral Flexion	0.9	(3.3)	2.2	(2.6)	4.1	(3.0)	12.5	**<0.001**
Trunk Backward Rotation	3.0	(6.3)	-3.1	(5.8)	-6.6	(6.1)	20.8	**<0.001**
**At 60 Knee Flexion**								
Knee Flexion	60.4	(0.1)	60.4	(0.1)	60.4	(0.2)	0.4	0.704
Knee Abduction	4.8	(4.9)	2.4	(4.0)	2.6	(4.3)	10.5	**0.002**
Knee Internal Rotation	3.1	(6.6)	2.6	(7.1)	2.8	(7.1)	0.2	0.730
Hip Flexion	34.2	(11.3)	34.5	(8.2)	37.6	(7.5)	3.2	0.082
Hip Adduction	5.1	(6.8)	10.4	(7.6)	11.0	(7.5)	42.3	**<0.001**
Hip External Rotation	7.9	(5.2)	4.3	(5.1)	5.0	(4.7)	32.7	**<0.001**
Pelvic Anterior Tilt	4.4	(9.3)	9.4	(6.2)	10.8	(6.0)	17.9	**<0.001**
Pelvic Drop	-3.0	(2.6)	1.1	(2.8)	1.4	(3.2)	51.2	**<0.001**
Pelvic Backward Rotation	1.2	(5.2)	-2.7	(5.4)	-3.8	(5.6)	13.9	**<0.001**
Trunk Flexion	8.9	(6.3)	3.9	(5.4)	7.6	(4.9)	6.4	**0.021**
Trunk Ipsilateral Flexion	1.0	(1.8)	1.9	(2.1)	2.2	(2.1)	3.7	**0.038**
Trunk Backward Rotation	3.9	(5.0)	-1.1	(5.5)	-1.1	(4.7)	14.8	**<0.001**

Results of the repeated measures ANOVA are presented with bold text indicating significance. SLS = Single leg squat; SD16 = step down from 16 cm; SD24 = step down from 24 cm.

**Table 2:** Results of post-hoc tests and effect size (ES) for each variable for each comparison at the two time points are presented with bold text indicating significance.

	SLS vs SD16		SLS vs SD24		SD16 vs SD24	
Movement	*P*	ES	*P*	ES	*P*	ES
**At Peak Knee Flexion**						
Knee Flexion	**<0.001**	1.8	0.778	0.3	**<0.001**	2.0
Knee Abduction	0.029	0.6	**<0.001**	1.0	0.238	0.3
Knee Internal Rotation	Not Tested					
Hip Flexion	0.114	0.5	**0.013**	0.7	**<0.001**	1.6
Hip Adduction	**0.003**	0.7	**<0.001**	1.2	**<0.001**	0.5
Hip External Rotation	**0.001**	0.7	0.062	0.3	**0.049**	0.4
Pelvic Anterior Tilt	**<0.001**	0.9	**<0.001**	1.3	**0.002**	0.6
Pelvic Drop	**<0.001**	1.8	**<0.001**	2.1	**0.036**	0.5
Pelvic Backward Rotation	0.189	0.5	**<0.001**	1.5	**0.001**	1.2
Trunk Flexion	**0.007**	1.3	0.158	0.7	**<0.001**	0.9
Trunk Ipsilateral Flexion	0.227	0.4	**0.001**	1.0	**0.024**	0.7
Trunk Backward Rotation	**0.004**	1.0	**<0.001**	1.6	**0.047**	0.6
**At 60 Knee Flexion**						
Knee Flexion	Not Tested					
Knee Abduction	**0.004**	0.6	**0.030**	0.5	1.000	0.1
Knee Internal Rotation	Not Tested					
Hip Flexion	Not Tested					
Hip Adduction	**<0.001**	0.7	**<0.001**	0.8	0.809	0.1
Hip External Rotation	**<0.001**	0.7	**<0.001**	0.6	0.399	0.1
Pelvic Anterior Tilt	**0.007**	0.6	**0.001**	0.8	0.092	0.2
Pelvic Drop	**<0.001**	1.5	**<0.001**	1.5	1.000	0.1
Pelvic Backward Rotation	**0.011**	0.7	**0.001**	0.9	0.582	0.2
Trunk Flexion	0.051	0.8	1.000	0.2	**<0.001**	0.7
Trunk Ipsilateral Flexion	0.338	0.5	0.082	0.6	1.000	0.1
Trunk Backward Rotation	**0.004**	0.9	**0.001**	1.0	1.000	0.0

SLS = Single leg squat; SD16 = step down from 16 cm; SD24 = step down from 24 cm.

**Table 3:** Pearson correlation coefficients (r) for each variable for each comparison at the two time points are presented with bold text indicating significance.

	SLS vs SD16		SLS vs SD24			SD16 vs SD24		
Movement	*r*	*P*		*r*	*P*		*r*	*P*
**At Peak Knee Flexion**								
Knee Flexion	0.61	0.063		0.51	0.192		0.76	**0.006**
Knee Abduction	0.69	**0.021**		0.82	**<0.001**		0.81	**<0.001**
Knee Internal Rotation	0.95	**<0.001**		0.92	**<0.001**		0.96	**<0.001**
Hip Flexion	0.73	**0.009**		0.76	**0.003**		0.85	**<0.001**
Hip Adduction	0.81	**<0.001**		0.86	**<0.001**		0.93	**<0.001**
Hip External Rotation	0.86	**<0.001**		0.91	**<0.001**		0.86	**<0.001**
Pelvic Anterior Tilt	0.90	**<0.001**		0.89	**<0.001**		0.90	**<0.001**
Pelvic Drop	0.56	0.117		0.69	**0.021**		0.84	**<0.001**
Pelvic Backward Rotation	0.62	0.057		0.57	0.105		0.57	0.105
Trunk Flexion	0.21	1.410		0.39	0.501		0.86	**<0.001**
Trunk Ipsilateral Flexion	0.66	**0.033**		0.69	**0.018**		0.66	**0.030**
Trunk Backward Rotation	0.58	0.090		0.45	0.309		0.68	**0.021**
**At 60 Knee Flexion**								
Knee Flexion	0.16	1.734		0.06	2.481		0.13	1.959
Knee Abduction	0.90	**<0.001**		0.83	**<0.001**		0.94	**<0.001**
Knee Internal Rotation	0.91	**<0.001**		0.88	**<0.001**		0.98	**<0.001**
Hip Flexion	0.84	**<0.001**		0.83	**<0.001**		0.93	**<0.001**
Hip Adduction	0.93	**<0.001**		0.92	**<0.001**		0.97	**<0.001**
Hip External Rotation	0.93	**<0.001**		0.94	**<0.001**		0.96	**<0.001**
Pelvic Anterior Tilt	0.86	**<0.001**		0.89	**<0.001**		0.94	**<0.001**
Pelvic Drop	0.76	**0.006**		0.71	**0.015**		0.96	**<0.001**
Pelvic Backward Rotation	0.70	**0.015**		0.75	**0.006**		0.85	**<0.001**
Trunk Flexion	0.34	0.705		0.43	0.390		0.93	**<0.001**
Trunk Ipsilateral Flexion	0.51	0.186		0.60	0.072		0.83	**<0.001**
Trunk Backward Rotation	0.61	0.060		0.68	**0.024**		0.82	**<0.001**

SLS = Single leg squat; SD16 = step down from 16 cm; SD24 = step down from 24 cm.

### Knee

The peak knee flexion angle was less in the SD16 task than in either the SLS or the SD24 task (p < 0.001). The knee was in more abduction at both peak knee flexion (p ≤ 0.029) and 60° of knee flexion in the SLS task than in either of the SD tasks (p ≤ 0.030). The knee was in internal rotation at both time points, and was not different between tasks.

The peak knee flexion angle was moderately correlated between the two step down tasks. The knee abduction angles had a moderate to excellent correlation between tasks at both time points (r = 0.69–0.94, p ≤ 0.021). The knee internal rotation angle had an excellent correlation between tasks at both time points (r = 0.88–0.98, p < 0.001).

### Hip

At peak knee flexion, the hip flexion angle and hip adduction angle were both greater in the SD24 task than in either the SLS or SD16 task (p ≤ 0.013). Also at peak knee flexion, the hip was in external rotation with less rotation in the SD16 task than in either the SLS or SD24 (p ≤ 0.049). Additionally, the hip adduction angle was less in the SLS task than either SD16 or SD24 at both peak knee flexion (p ≤ 0.003) and 60° of knee flexion (p < 0.001). At 60° of knee flexion, the hip external rotation was greater in the SLS task than in either step down task.

At peak knee flexion and at 60° of knee flexion, hip flexion, adduction, and external rotation were each correlated between the three tasks. The correlations were generally lower at peak knee flexion (r = 0.73–0.93, p ≤ 0.009) than at 60° of knee flexion (r = 0.83–0.97, p < 0.001).

### Pelvis

At peak knee flexion, the pelvis was anteriorly tilted more, rotated forward more, and in greater contralateral pelvic drop in the SD24 task than in either the SLS or the SD16 task (p ≤ 0.036). At both time points, the pelvis was anteriorly tilted less and in less contralateral pelvic drop in the SLS task than either step down task (p ≤ 0.007). At 60° of knee flexion, there was less forward pelvic rotation (as the pelvis was actually rotated backward) during the SLS task than either SD task (p ≤ 0.011).

At peak knee flexion, only the anterior pelvic tilt had an excellent correlation between all tasks (r = 0.89–0.90, p < 0.001). Pelvic drop in SD24 was correlated with both SLS and SD16 (r = 0.69, p = 0.021; r = 0.84, p < 0.001, respectively). Pelvic rotation, however, was not correlated between tasks at peak knee flexion (r = 0.57–0.62, p > 0.057). At 60° of knee flexion, pelvic angles in all three directions were correlated between the three tasks. The correlations between SD16 and SD24 were generally higher than the correlations between SLS and either step down task (r = 0.85–0.94 vs. 0.70–0.89, respectively).

### Trunk

The trunk was in less flexion in the SD16 than in the SD24 task at both time points (p < 0.001), and less than the SLS task at peak knee flexion (p = 0.007). At both time points, the trunk was rotated backward more in the SLS task than both step down tasks. At peak knee flexion, the trunk was more laterally flexed toward the stance leg (ipsilateral flexion) and rotated forward in the SD24 task than either the SD16 or the SLS task (p ≤ 0.024).

The trunk angles in each of the three directions were correlated between SD16 and SD24 at both peak knee flexion (r = 0.66–0.86, p ≤ 0.030) and at 60° of knee flexion (r = 0.82–0.93, p < 0.001). At peak knee flexion, ipsilateral trunk flexion was correlated between SLS and the two step down tasks (r = 0.66–0.69, p ≤ 0.033). At 60° of knee flexion, trunk backward rotation was correlated between SLS and SD24 (r = 0.68, p = 0.024).

## Discussion

The single leg squat task and the step down task are commonly used as a functional screening test for the lower extremity. This study investigated if differences exist in knee, hip, pelvis, and trunk angles between three weight-bearing tasks and the correlation between the angles measured with each task. The general movement pattern during the descent phase of each task was hip and knee flexion, combined with initial anterior pelvic tilt and trunk flexion. In the frontal plane, knee abduction was accompanied by hip adduction and ipsilateral trunk flexion. In the transverse plane, the consistent motion between all three tasks was knee internal rotation. The differences were primarily in the magnitude of these movements. The movement patterns that were starkly different between tasks were the motion of the pelvis in the frontal and transverse planes, and the trunk in the transverse plane. During the single leg squat task, the pelvis was maintained in a hiked position and backward rotation while during the step down task at each height, there consistently was pelvic drop and forward rotation. Additionally, the trunk was rotated backward during the single leg squat task while it was rotated forward during the step down task.

The results of this study highlight the importance of three things: 1) task instruction, 2) point of comparison, and 3) exercise selection. First, the instructions for the step down and for the single leg squat tasks are fundamentally different and result in different movement patterns. The goal of the single leg squat task is maximum knee flexion while maintaining balance and keeping the non-stance limb off the ground. Hiking the pelvis and minimizing stance hip adduction would help keep the non-stance limb off the ground. The goal of the step down task, as performed in this study and elsewhere [9,23,33,47,48], is to touch the contralateral heel to the ground, and then return to standing. This could be achieved with less hip and knee flexion simply by adducting the stance hip and dropping the pelvis. Thus, the increased hip adduction observed during the step down task was a result of the task and not necessarily a reflection of the person’s ability to control the movement.

Second, the point of comparison used for analysis between tasks affected the results. Specifically, SD16 seems very different from SD24 when compared at peak knee flexion. However, when compared at 60° of knee flexion, only the sagittal trunk angle was different between step down tasks. This finding indicates that, for a range of step heights, the same movement pattern is used for the step down task. This finding also suggests that comparisons can be made across studies with slightly different step heights, if analyzed at the same knee angle. Furthermore, it may not be necessary to test at multiple step heights if the study question is about joint angles at a particular point in the cycle. Conversely, the single leg squat task continued to be different from the step down tasks at both time points. This finding, again, indicates that single leg squat and step down use different movement patterns. These patterns primarily differ in the frontal and transverse planes at 60° of knee flexion, with increased knee abduction and hip external rotation, but decreased hip adduction, in the single leg squat task compared to the step down task. There was also less anterior pelvic tilt, more pelvic hiking and backward rotation, and more trunk backward rotation in the single leg squat compared to the step down.

While there were differences in the angles between the three tasks, especially at peak knee flexion, the angles had a moderate to excellent relationship between tasks. Between SD16 and SD24, all angles were correlated at both time points except for pelvic backward rotation at peak knee flexion and knee flexion at 60° of knee flexion. The correlations between the angles during the single leg squat task and during the step down tasks were lower than within the step down tasks. At peak knee flexion, knee flexion, pelvic backward rotation, trunk flexion, and trunk backward rotation were not correlated between SLS and either step down task. Additionally, pelvic drop was not correlated between SLS and SD16. At 60° of knee flexion, trunk flexion and ipsilateral flexion were not correlated between SLS and either step down task. Trunk backward rotation was not correlated between SLS and SD16. The excellent correlation of knee and hip angles between the three tasks indicate that, while the tasks were different, similar information about the knee and hip was gained from each of these tasks. This finding indicates that different tasks may not be necessary if the focus of the assessment is on the kinematics of the knee and hip.

Third, the kinematic differences noted between tasks are important when selecting exercises for a patient. The single leg squat task was performed with more knee abduction (dynamic valgus) while the step down task was performed with more hip adduction. While the rehabilitation goal of the exercise would likely be to reduce both knee abduction and hip adduction, the exercises may have varying degrees of difficulty and could be used in an exercise progression. For example, a single leg squat task might be a more appropriate exercise for a patient with femoroacetabular impingement (FAI), a structural hip condition exacerbated by combined hip adduction and flexion [49–52]. Similarly, for a patient with PFP, the step down task might be less appropriate as increased hip adduction has been linked to PFP [25,31,53]. In return, the step down task might be more appropriate for a patient with a history of ACL injury, as increased knee abduction may contribute to this injury [54,55].

Our data are within the ranges of data in the literature for these movement tasks in healthy participants. For the single leg squat, there is significant variability in the literature. Knee flexion angles ranges from under 70° [56] to over 100° [18,20,57–59]. Our SD24 data do match some of data in the literature for the hip and knee during a step down task [32,60]. Differences in instructions, squat depth/step height and point of analysis, as well as differences in definitions of segmental coordinate systems, may contribute to the wide ranges.

There are limitations due to the design of this study. We selected healthy asymptomatic participants to better understand the normal kinematic patterns during the three tasks. Determining the normal kinematic differences between tasks will better inform clinicians and researchers when interpreting differences detected in populations with movement impairments. Although studies have noted differences in movement patterns between males and females [25,30,57,59], we did not analyze the sexes separately. As this study used a repeated measures design, we surmised that the within participants changes would not be different, and therefore, would not be affected by the sex of the participants. The anterior position of the non-stance limb during the single leg squat is different from positioning used in some other studies where the non-stance limb is held posterior to the stance limb [12,13,20,59]. This anterior position was used because the non-stance limb is also positioned anteriorly in the step down task. The positioning of the non-stance limb may affect the movement pattern. It should also be noted that cues were not given to “correct” performance during or after completion of the task as we were interested in capturing the natural performance of the task in healthy participants. Verbal cues can significantly change the performance of the task [36]. Another limitation is that we did not randomize task order, as this was part of a larger clinical study which used this progression of tasks.

## Conclusions

The results from this study indicate the single leg squat task and step down task result in different lower extremity, pelvis, and trunk kinematics. The angles in all directions evaluated at the knee and hip had a moderate to excellent correlation, while the angles at the pelvis and trunk were less well correlated. The findings from this study provide the clinician with more information when selecting the most appropriate task or exercise for patients with lower extremity movement dysfunction.
